# Association between EuroSCORE II and postoperative delirium in cardiac surgery patients: a prospective cross-sectional study

**DOI:** 10.1097/MS9.0000000000004028

**Published:** 2025-10-13

**Authors:** Khalid Maudood Siddiqui, Basma Andrabi, Muhammad Saad Yousuf

**Affiliations:** Department of Anaesthesiology, The Aga Khan University, Karachi, Pakistan

**Keywords:** CAM-ICU, cardiac surgery, cognitive outcomes, EuroSCORE II, postoperative delirium, risk stratification

## Abstract

**Background:**

Postoperative delirium (POD) is a significant neuropsychiatric issue after cardiac surgery, linked to increased morbidity, lengthened hospitalization, and adverse outcomes. EuroSCORE II is a validated tool for predicting surgical mortality, but its predictive utility for POD remains underexplored. This study focused on assessing the relationship between preoperative EuroSCORE II and the incidence of POD in adult patients undergoing elective cardiac surgery.

**Materials and methods:**

A prospective cross-sectional study was conducted, including 270 patients aged between 18 and 75 years scheduled for elective coronary artery bypass grafting, mitral valve replacement, or aortic valve replacement. Preoperative EuroSCORE II was calculated, and patients were stratified into four risk categories. POD was assessed using the Confusion Assessment Method for intensive care unit every 12 hours for 48 hours postoperatively. Models using logistic regression were employed to determine independent predictors of POD.

**Results:**

POD incidence was 4.3%. Patients in the moderate-to-high EuroSCORE II category had a significantly higher incidence of POD as compared to low-risk groups (*P* < 0.05). A higher EuroSCORE II was associated with greater delirium severity at all time points. Age was an independent predictor of POD (adjusted odds ratio = 1.19; 95% confidence interval: 1.09–1.32; *P* < 0.001), while diabetes and hypertension, although prevalent in higher-risk groups, were not independently predictive. Intraoperative variables such as cardio-pulmonary bypass, aortic cross-clamp times, and duration of surgery showed no significant association with POD.

**Conclusion:**

EuroSCORE II is significantly associated with both the incidence and severity of POD after cardiac surgery. It may be a useful component of preoperative risk stratification models for cognitive outcomes. However, its standalone predictive power is limited, and future models should integrate cognitive screening and frailty assessment to enhance accuracy.

## Introduction

Patients undergoing heart surgery are at significant risk of developing delirium, a prevalent psychiatric illness among those who have cardiac surgery, with a prevalence of 17.3%–23.5%^[1–3]^. Delirium is closely associated with increased mortality, cognitive impairment, loss of functional independence, and higher hospitalization costs^[1–3]^. Delirium also leads to prolonged hospital and intensive care unit (ICU) stays, increasing morbidity, mortality, and healthcare costs. The condition also negatively affects postoperative rehabilitation, quality of life, and often results in social dependence^[4,5]^.

**HIGHLIGHTS**

- Patients with higher EuroSCORE II scores had significantly greater delirium severity, especially within the first 12 hours postoperatively.
- EuroSCORE II may serve as a useful preoperative stratification tool for cognitive outcomes, but its standalone predictive power for delirium is limited.
- Integrating cognitive screening and frailty assessments alongside EuroSCORE II could improve predictive accuracy and guide early intervention strategies for high-risk patients.

Delirium poses significant diagnostic challenges due to its fluctuating nature and varied presentations, requiring a comprehensive approach for early recognition and management[6]. Postoperative cognitive disorders are common complications following cardiac surgery. The incidence of postoperative delirium (POD) has ranged from 15% to 55% depending on risk factors, including advanced age, carotid artery disease, anemia, prolonged cardiopulmonary bypass (CPB) time, and extended ICU stay^[7,8]^.

In order to make informed medical decisions, patients undergoing heart surgery typically need risk assessment and prediction tools. The European System for Cardiac Operative Risk Evaluation II (EuroSCORE II) is one of the most frequently utilized prediction tools for risk stratification, which includes 18 independent variables. It is employed to evaluate the risk involved with heart surgery.

Therefore, recognizing the numerous risk factors associated with delirium, including patient age and preexisting conditions, is crucial. Previous literature has identified risk factors associated with delirium, including patients with advanced age, higher EuroSCORE II, and longer aortic cross-clamp (ACC) time^[9,10]^.

The main objectives of this study are to evaluate the association between preoperative EuroSCORE II and the incidence of POD in patients undergoing cardiac surgery. Specifically, the study aims to determine whether higher EuroSCORE II values are predictive of an increased risk of developing delirium in the postoperative period. Establishing such a relationship would support the utility of EuroSCORE II as a risk stratification tool not only for mortality and morbidity but also for neurocognitive complications such as delirium.

The primary outcome of this study is to determine the incidence of POD in relation to preoperative EuroSCORE II values, and secondary outcomes considered as the identification of specific components within EuroSCORE II that may be most strongly associated with delirium and the analysis of the duration and severity of delirium in relation to EuroSCORE II scores.

## Methods

This prospective cross-sectional study was carried out in a single tertiary care center. Ethical approval for this study (ERC #2021-6153-19008) was provided by the Ethical Review Committee of the same institution on August 31, 2021.

**Figure 1.**
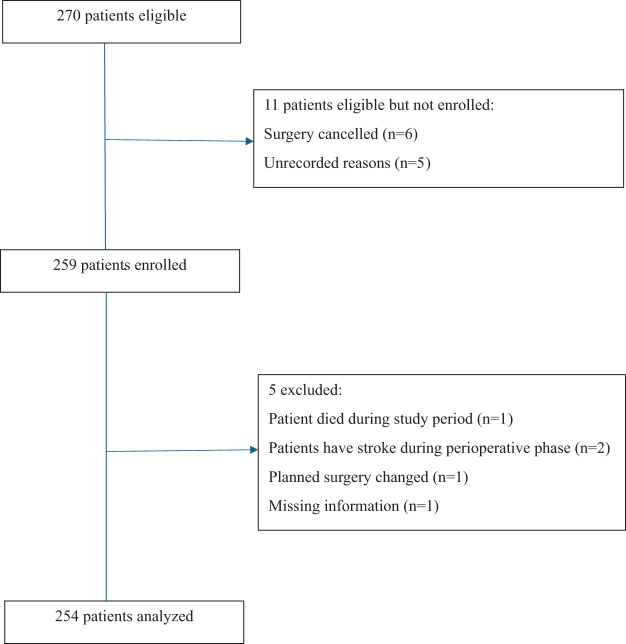
Flowchart of the study.

A total of 270 patients aged between 18 and 75 years of either gender with American Society of Anesthesiologists (ASA) physical status classification I–IV, scheduled for elective cardiac procedures such as coronary artery bypass grafting, mitral valve replacement, and aortic valve replacement, were included. Patients with emergency surgeries, those with a history of stroke within the last 6 weeks, previous history of delirium or psychiatric illness, those receiving psychiatric medications, or undergoing reoperative cardiac procedures were excluded. Written informed consent was obtained from all participants before enrolment in the study. The flow diagram of the study is shown in Figure 1.

The study focused on patients undergoing elective cardiac surgeries and was carried out across three perioperative settings: the preoperative area, the operating rooms, and the Cardiac Intensive Care Unit (CICU).

Sample size calculation was based on the findings of Sabol *et al*[11], who reported a 20.8% incidence of POD in cardiac surgery patients. Assuming a similar incidence in the local population and using a 7.96% margin of error with a 95% confidence interval, a minimum of 270 patients were required. A non-probability consecutive sampling technique was used to recruit patients meeting the eligibility criteria.

Patients’ gender, age, height, weight, body mass index (BMI), comorbidities (such as diabetes, hypertension, and smoking), ASA classification, and clinical diagnosis were among the baseline demographic and clinical information gathered prior to surgery. The EuroSCORE II was calculated for each patient using the official calculator from the European System for Cardiac Operative Risk Evaluation website (www.euroscore.org).

All patients received standard perioperative care according to local procedural protocols of cardiac surgery. Intraoperative variables, including the duration of surgery, ACC time, CPB duration, lowest temperature during CPB, and blood transfusion requirements, were extracted from anesthesia charts and recorded.

Fast-track extubation was performed postoperatively, and all patients were monitored for signs of POD using the Confusion Assessment Method for the Intensive Care Unit (CAM-ICU) tool. The CAM-ICU score was calculated at 12-hour intervals for 48 hours postoperatively. A score-based version, CAM-ICU-7, assigned scores to each feature to assess delirium severity, with scores ranging from 0 to 7 (0–2: No delirium, 3–5: Mild to moderate delirium, and 6–7: Severe delirium).

The collection of data was carried out solely by the coprincipal investigator, who ensured that each patient’s information was accurately and consistently recorded. All collected data were treated with strict confidentiality. Hardcopy forms were securely stored under lock and key, while digital records were maintained in a password-protected database.

This study is reported in line with the STROBE cross-sectional reporting guidelines[12] and the STROCSS guideline[13].

### Data analysis

All data were analyzed in SPSS version 22. The normality of quantitative variables was reviewed by Q-Q plot and Kolmogorov–Smirnov. Mean, standard deviation, median, and IQR were computed as per the normality assumption for age, duration of surgery, ACC, CPB duration, POD, and EuroSCORE II scores. Frequency and percentage were computed for qualitative variables like sex, types of procedure, delirium (yes/no), and EuroSCORE II score categories (very low risk [<1; *n* = 2], low risk [1–2.99; *n* = 136], moderate risk [3–4.99; *n* = 97], and high risk [>5; *n* = 19]). After recruitment and calculation of EuroSCORE II, patients were subsequently categorized into two groups for analysis: Low risk (EuroSCORE II <3; *n* = 138, 54.3%) and Moderate-to-high risk (EuroSCORE II ≥3; *n* = 116, 45.7%). This categorization was applied during statistical analysis to compare baseline characteristics and the incidence of delirium. The association of EuroSCORE II score and delirium was assessed by univariate logistic regression, and the crude odds ratio was reported with 95% CI. Multivariable logistic regression was also applied to adjust for the effect of confounding variables on the association of EuroSCORE II score and delirium. In order to identify independent risk factors, variables considered clinically significant and those related to the outcome with a *P*-value <0.05 in the univariate analyses were added to a multiple logistic regression model for delirium.

## Results

Patients in the moderate-to-high EuroSCORE II group had a higher mean age (59.4 ± 12.0 years) compared with the low-risk group (54.7 ± 10.5 years, *P* = 0.001). Gender distribution was similar: males comprised 77.5% in the low-risk and 77.6% in the higher-risk group. BMI was comparable (26.4 vs 27.1 kg/m^2^, *P* = 0.233). Diabetes and hypertension were significantly more common in the higher-risk group (73.3% vs 47.1%, and 72.4% vs 50.0%, both *P* < 0.001). No significant differences were found in gender (*P* = 1.000), history of smoking (*P* = 0.101), or type of planned cardiac procedure. The baseline demographic and clinical characteristics of these groups are presented in Table 1.

**Table 1:** Comparison of baseline characteristics and clinical parameters between low and moderate-to-high EuroSCORE II groups

Dependent: EuroSCORE II	Low	Moderate-to-high	*P*-value
Age (years), mean (SD)	54.7 (10.5)	59.4 (12.0)	0.001
Gender			
Male	107 (77.5)	90 (77.6)	1.000
Female	31 (22.5)	26 (22.4)	
BMI, mean (SD)	26.4 (4.7)	27.1 (4.6)	0.233
Comorbidities			
Diabetes mellitus			
No	73 (52.9)	31 (26.7)	<0.001
Yes	65 (47.1)	85 (73.3)	
Hypertension			
No	69 (50.0)	32 (27.6)	<0.001
Yes	69 (50.0)	84 (72.4)	
History of smoking			
No	122 (88.4)	93 (80.2)	0.101
Yes	16 (11.6)	23 (19.8)	
Planned surgical procedure			
CABG	108 (78.3)	87 (75.0)	0.227
AVR	9 (6.5)	8 (6.9)	
CABG + AVR	2 (1.4)	4 (3.4)	
CABG + MVR	1 (0.7)	2 (1.7)	
DVR	2 (1.4)	7 (6.0)	
MVR	16 (11.6)	8 (6.9)	
Duration of surgery (minutes), mean (SD)	256.3 (52.3)	253.7 (65.3)	0.718
ACC time, mean (SD)	63.9 (31.8)	66.0 (34.3)	0.609
CPB time, mean (SD)	96.7 (40.2)	96.6 (40.5)	0.986
Minimum temperature during CPB, mean (SD)	32.3 (0.8)	32.0 (0.8)	0.006
Blood transfusion, mean (SD)	0.6 (0.7)	0.9 (0.8)	0.003

Apply chi-square statistic, consider statistically significant if the *P*-value is <0.05.

ACC, aortic cross-clamp; AVR, aortic valve replacement; BMI, body mass index; CABG, coronary artery bypass grafting; CPB, cardiopulmonary bypass; DVR, double valve replacement; MVR, mitral valve replacement.

In multivariable analysis, diabetes mellitus, hypertension, lower minimum temperature during CPB, and greater blood transfusion volume were significant independent predictors of higher EuroSCORE II. Age was significant only in univariable analysis. Gender, BMI, history of smoking, duration of surgery, ACC, and CBP times did not show statistically significant associations with EuroSCORE II (Table 2).

**Table 2:** Univariable and multivariable multinomial logistic regression analysis for predictors of EuroSCORE II

Dependent: EuroSCORE II	Univariable OR (95% CI)	Multivariable AOR (95% CI)
Age (years)	1.04 (1.02–1.07, *P* = 0.001)	1.02 (0.99–1.05, *P* = 0.156)
Sex		
Male (Ref)	–	–
Female	1.00 (0.55–1.80, *P* = 0.992)	
BMI	1.03 (0.98–1.09, *P* = 0.233)	1.01 (0.94–1.07, *P* = 0.856)
Comorbidities		
Diabetes mellitus	3.08 (1.83–5.28, *P* < 0.001)	2.52 (1.39–4.64, *P* = 0.003)
Hypertension	2.62 (1.56–4.48, *P* < 0.001)	2.05 (1.12–3.80, *P* = 0.021)
History of smoking	1.89 (0.95–3.83, *P* = 0.073)	2.18 (0.99–4.94, *P* = 0.055)
Duration of surgery (minutes)	1.00 (0.99–1.00, *P* = 0.717)	1.00 (0.99–1.00, *P* = 0.525)
ACC time	1.00 (0.99–1.01, *P* = 0.608)	–
CPB time	1.00 (0.99–1.01, *P* = 0.986)	–
Minimum temperature during CPB	0.63 (0.43–0.87, *P* = 0.010)	0.54 (0.35–0.81, *P* = 0.004)
Blood transfusion	1.69 (1.19–2.43, *P* = 0.004)	1.56 (1.05–2.36, *P* = 0.031)

Univariate and multivariate analyses were applied; *P*-values <0.05 were considered statistically significant.

AOR, adjusted odds ratio; CPB, cardiopulmonary bypass; OR, odds ratio.

Both univariable and multivariable analyses showed that increasing age was a significant independent predictor of POD (AOR: 1.19, *P* < 0.001). Other variables, including gender, BMI, diabetes, hypertension, smoking, duration of surgery, ACC and CPB time, minimum temperature, and blood transfusion, did not show statistically significant associations (Table 3).

**Table 3:** Univariable and multivariable multinomial logistic regression analysis for predictors of delirium

Dependent: Delirium	Univariable OR (95% CI)	Multivariable AOR (95% CI)
Age (years)	1.17 (1.08-1.28, *P* < 0.001)	1.19 (1.09–1.32, *P* < 0.001)
Gender		
Male (Ref.)	–	–
Female	1.31 (0.28–4.71, *P* = 0.695)	
BMI	0.95 (0.82–1.08, *P* = 0.445)	–
Comorbidities		
Diabetes mellitus		
No (Ref.)	–	–
Yes	7.36 (1.38–136.08, *P* = 0.059)	5.79 (0.83–132.58, *P* = 0.145)
Hypertension		
No (Ref.)	–	–
Yes	3.09 (0.78–20.58, *P* = 0.154)	1.92 (0.40–15.09, *P* = 0.461)
History of smoking		
No-Ref	–	–
Yes	1.24 (0.18–5.04, *P* = 0.791)	–
Duration of surgery (mins)	1.00 (0.98–1.01, *P* = 0.451)	–
ACC time (mins)	1.00 (0.99–1.02, *P* = 0.564)	–
CPB time (mins)	1.00 (0.99–1.02, *P* = 0.529)	–
Minimum temperature during CPB	0.81 (0.44–1.66, *P* = 0.560)	–
Blood transfusion	1.37 (0.61–2.81, *P* = 0.414)	–

Univariate and multivariate analyses were applied; *P*-values <0.05 were considered statistically significant.

AOR, adjusted odds ratio; OR, odds ratio.

Patients with very low and low EuroSCORE II had no incidence of POD. In the moderate-risk group, 4.1% developed delirium, while the high-risk group showed the highest incidence at 36.8%. Overall, a higher EuroSCORE II was significantly associated with increased delirium occurrence (*P* < 0.05). Low-risk patients consistently showed the highest proportion without delirium, indicating a strong correlation between surgical risk and POD incidence (Table 4).

**Table 4:** Association between the EuroSCORE II and delirium

EuroSCORE II	Postoperative delirium parameters		*P*-value
	No *n* (%)	Yes *n* (%)	
Very low	2 (100)	0 (0)	<0.05
Low	136 (100)	0 (0)	
Moderate	93 (95.9)	4 (4.1)	
High	12 (63.2)	7 (36.8)	

Chi-square tests were applied; *P* values <0.05 were considered statistically significant.

A significant association was observed between EuroSCORE II and delirium severity at all time points (12, 24, 36, and 48 hours; *P* < 0.05). Patients with high EuroSCORE II were more likely to experience moderate to severe delirium, especially at 12 hours. A significant association (*P* < 0.05) was found between higher CAM-ICU scores (≥3) and delirium at all postoperative time points. Delirium incidence was highest with CAM-ICU scores of 6–7, especially at 12 hours. Scores of 0–2 consistently indicated no or very low risk of delirium (Table 5).

**Table 5:** CAM-ICU score on delirium in different timelines

	Postoperative delirium parameters		*P*-value
	No *n* (%)	Yes *n* (%)	
CAM-ICU score at 12 hrs
0–2	229 (100)	0 (0)	<0.05
3–5	14 (100)	0 (0)	
6–7	0 (0)	11 (100)	
CAM-ICU score at 24 hrs			<0.05
0–2	236 (96.7)	8 (3.3)	
3–5	7 (70)	3 (30)	
6–7	0 (0)	0 (0)	
CAM-ICU score at 36 hrs			<0.05
0–2	241 (96.4)	9 (3.6)	
3–5	2 (50)	2 (50)	
6–7	0 (0)	0 (0)	
CAM-ICU score at 48 hrs			<0.05
0–2	241 (96.4)	9 (3.6)	
3–5	2 (50)	2 (50)	
6–7	0 (0)	0 (0)	

## Discussion

Our study aimed to assess the association between the EuroSCORE II and the incidence of POD in adult patients undergoing elective cardiac surgery. In our study overall, the incidence of POD after cardiac surgery was 4.3%. Neurocognitive deficits following heart surgery have a complex etiology that includes known patient risk factors as well as perioperative insults[14]. Therefore, POD remains a major neuropsychiatric complication following cardiac surgery, particularly among elderly patients[15].

Although the incidence of POD in our study (4.3%) was lower than that reported in previous cardiac surgery cohorts, several factors may account for this difference. First, our study exclusively included elective surgical cases, whereas emergency and high-complexity procedures, which carry a higher delirium burden, were excluded. Second, the study population was relatively younger (mean age 56.9 years) compared with Western cohorts, where advanced age is a dominant risk factor for POD. Third, stringent exclusion criteria, such as prior psychiatric illness, recent stroke, or reoperations, removed patients with pre-existing vulnerabilities known to elevate delirium risk. Fourth, all patients were managed under standardized perioperative and CICU protocols, including early extubation, routine use of CAM-ICU monitoring, and optimized hemodynamic management, which are recognized strategies for mitigating delirium incidence. Importantly, while the absolute incidence was lower, the relative association between higher EuroSCORE II and POD remained consistent, supporting the validity of our findings.

While EuroSCORE II is conventionally used to estimate in-hospital mortality risk, its utility in predicting neurocognitive complications like delirium has been sparsely studied. Our findings suggest that EuroSCORE II may serve as a clinically meaningful predictor of POD, especially when interpreted alongside age and comorbid burden.

Several studies have previously explored the risk factors associated with POD. Among the most consistent predictors are advanced age, cognitive impairment, comorbidities such as diabetes, hypertension, and complex intraoperative variables like prolonged CPB duration^[16,17]^. This study reinforces the role of age as an independent and statistically significant predictor of delirium, consistent with extensive prior evidence^[18–20]^.

Interestingly, although comorbid conditions such as diabetes mellitus and hypertension were more frequent in higher EuroSCORE II strata, their individual predictive value for POD did not reach statistical significance in the multivariable model. This finding mirrors prior studies suggesting that while these conditions contribute to overall patient vulnerability, their isolated influence on delirium risk may be moderated by other physiological or perioperative factors^[21,22]^.

Moreover, EuroSCORE II, as a composite metric, includes age, renal function, ejection fraction, and surgical complexity, all of which have been implicated in delirium pathophysiology. Our observation of its significant correlation with delirium severity at multiple postoperative time points supports its potential as an early delirium risk stratification tool. This agrees with findings by Kazmierski *et al*[23], who observed that higher EuroSCORE II values were associated with increased POD incidence. However, some studies have criticized the EuroSCORE II’s limited sensitivity in predicting neurological outcomes compared to specialized cognitive screening tools or frailty indices^[24–26]^.

Intraoperative factors, including aortic ACC, CPB duration, surgical time, and lowest temperature during bypass, did not show significant associations with POD in our study, which is also previously observed by Gosselt *et al*[27]. Prior literature offers some contradictory findings in this domain. O’Neal *et al*[17] and Guenther *et al*[28] suggest a link between prolonged CPB and delirium, while others, including Cereghetti *et al*[21] and Deiner and Silverstein[29], emphasized that predisposing factors often outweigh procedural duration in determining POD outcomes. The variability across studies may reflect differences in anesthetic protocols, intraoperative cerebral protection strategies, and institutional practices.

POD is now recognized not merely as a transient event but as a marker of acute brain failure, potentially accelerating long-term cognitive decline. The association of higher EuroSCORE II values with early-onset POD, especially within the first 12 hours post-extubation, highlights a window for preventive strategies. Given that EuroSCORE II is routinely calculated preoperatively, its integration into delirium prevention algorithms could enable timely intervention for high-risk individuals.

Patho-physiologically, POD is thought to result from neuroinflammation, oxidative stress, neurotransmitter imbalance, and cerebral hypoperfusion; all of which may be exacerbated in individuals with poor preoperative physiological reserves or high surgical risk scores[30]. EuroSCORE II, while not designed to reflect cerebral vulnerability per se, may capture systemic fragility that predisposes to neurocognitive complications.

It is important to recognize that not all studies support the utility of EuroSCORE II in predicting delirium. Raats *et al*[31] and Segernäs *et al*[32] found that specific cognitive screening tools, such as the Statistical Manual of Mental Disorders or ICD-10 and Mini-Mental State Examination, outperformed surgical risk scores in predicting POD.

Moreover, some authors argue that delirium should be viewed through a geriatric lens, requiring multidimensional assessments that incorporate cognition, function, mood, and resilience^[33,34]^. Therefore, while EuroSCORE II adds value, it should be supplemented by dedicated cognitive risk assessments like the CAM-ICU or rapid clinical test for delirium (4AT)[35], especially in older patients.

Strengths of this study include its prospective design, standardized data collection by a dedicated investigator, and use of the validated CAM-ICU tool for serial delirium assessment. The focus on consecutive elective cardiac surgery patients enhances the internal validity, while the 48-hour observation window captures the typical peak period of POD onset. Moreover, the study employed both univariable and multivariable logistic regression analyses, allowing for a more robust evaluation of associations while adjusting for potential confounders. By linking EuroSCORE II to both delirium incidence and severity, the findings provide a clinically actionable insight for preoperative planning. The exclusion of patients undergoing emergency surgeries, those with a recent stroke, previous history of delirium or psychiatric illness, those receiving psychiatric medications, and patients undergoing reoperative cardiac procedures was intended to ensure a homogeneous study population and to minimize confounding factors independently associated with a high risk of POD. Including such patients could have introduced significant bias, as their baseline vulnerability to delirium is well established and might have overshadowed the predictive role of EuroSCORE II, which was the primary focus of our analysis and strengthens the validity of our study.

However, several limitations must be acknowledged. First, the study was conducted at a single tertiary care center, limiting generalizability to broader populations with different perioperative practices. Second, preoperative cognitive status was not formally assessed, which is a known independent predictor of POD. This omission may have attenuated the detection of certain risk relationships. Third, the relatively small number of delirium cases, especially in the severe category, may have underpowered the analysis for detecting subtler effects of some variables. Fourth, while the CAM-ICU is validated and widely used, hypoactive delirium can be underdiagnosed, leading to potential outcome misclassification. Lastly, sedative and analgesic dosing regimens were not adjusted for in the analysis. Since benzodiazepines and alcohol consumption are established risk factors for delirium, their influence may have introduced residual confounding, and accounting for them in future studies would strengthen risk stratification. However, due to resource and data limitations, systematic screening, particularly for alcohol intake, could not be incorporated into the present study. We also acknowledged it as one of the limitations and highlighted it as an area for future investigation. Future studies should aim to validate our findings in multicenter cohorts and examine the added value of combining EuroSCORE II with cognitive and frailty assessments. The development of composite prediction models tailored specifically for POD could significantly enhance preoperative risk stratification and clinical decision-making. Moreover, randomized controlled trials evaluating delirium prevention strategies (e.g. multimodal reorientation, pharmacologic prophylaxis) in high EuroSCORE II patients could provide critical evidence to shift practice paradigms. Investigating biomarkers of neuroinflammation, or intraoperative cerebral oximetry monitoring, could also open new frontiers in delirium research.

### Conclusion

Prediction of delirium remains challenging due to its multifactorial nature; the current information does not allow for complete accuracy with EuroSCORE II. These findings suggest that EuroSCORE II could be an applicable preoperative tool to identify patients at higher risk for POD, enabling clinicians to implement targeted preventive strategies. However, its predictive value is stronger in higher-risk groups and may not be sufficient as a standalone predictor across all risk categories. Therefore, recognizing the risk factors identified in this study can aid in the early spotting of patients at high risk.

## Data Availability

The data associated with the paper are available from the corresponding author on reasonable request.

## Research registration unique identifying number (UIN)

The study is registered with Clinicaltrials.gov under NCT05218720.
